# Supplementary material for: Seminoma and Embryonal Carcinoma Footprints Identified by Analysis of Integrated Genome-Wide Epigenetic and Expression Profiles of Germ Cell Cancer Cell Lines
Source: PLoS One. 2014 Jun 2;9(6):e98330. doi: 10.1371/journal.pone.0098330 (PMC4041891; doi:10.1371/journal.pone.0098330)
Supplement: File S1 — ZIP file containing DMRforPairs output for significant regions. Please start from the html files. (ZIP) [file pone.0098330.s008.zip › figures/29059.pdf]

RegionID: 29059, chr3:49026934−49027409−M_values

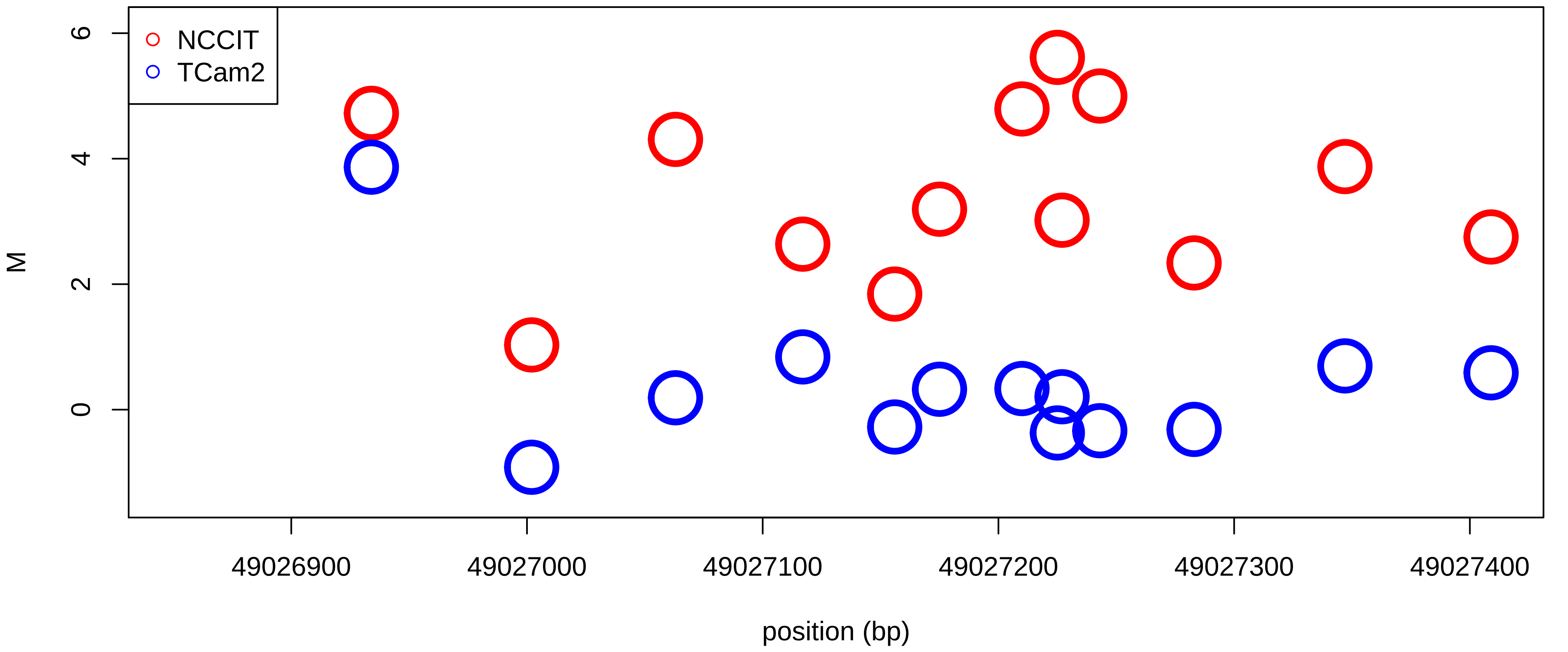

RegionID: 29059, chr3:49026934−49027409−Beta_values

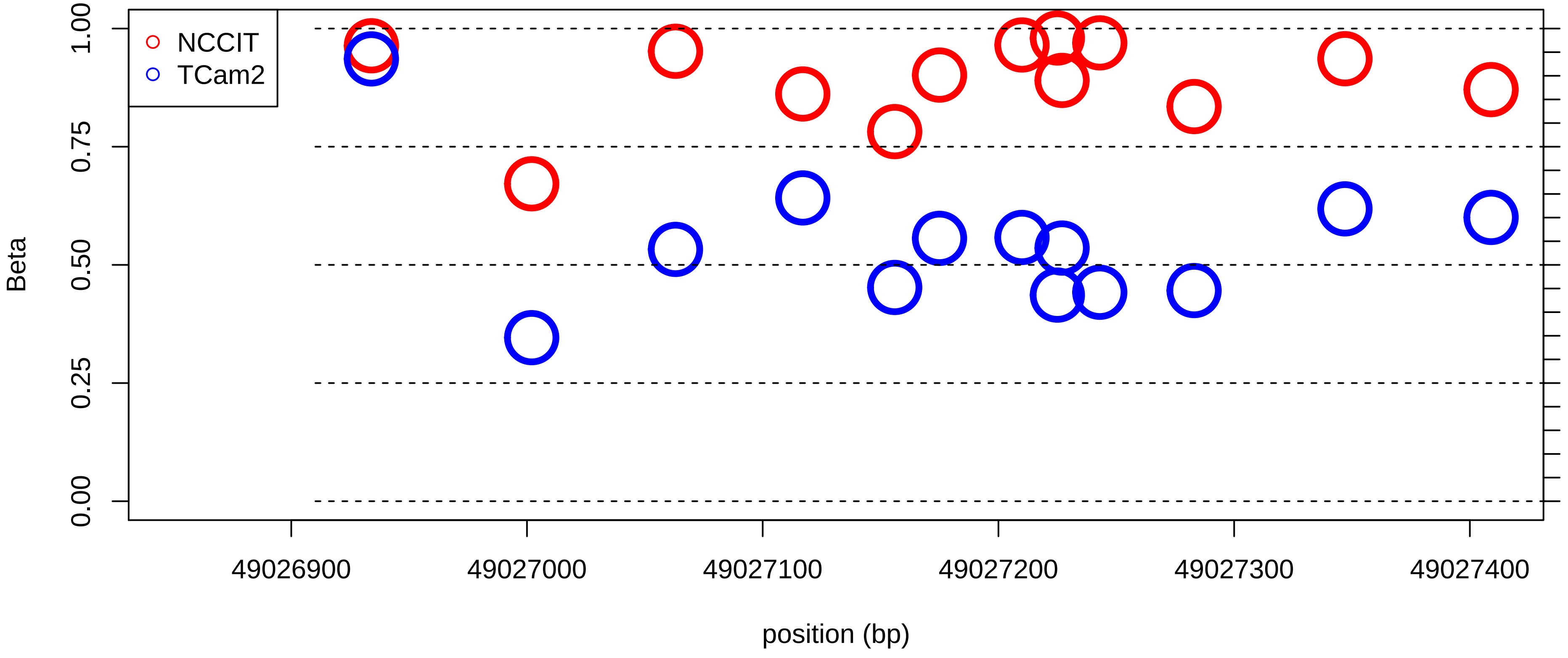

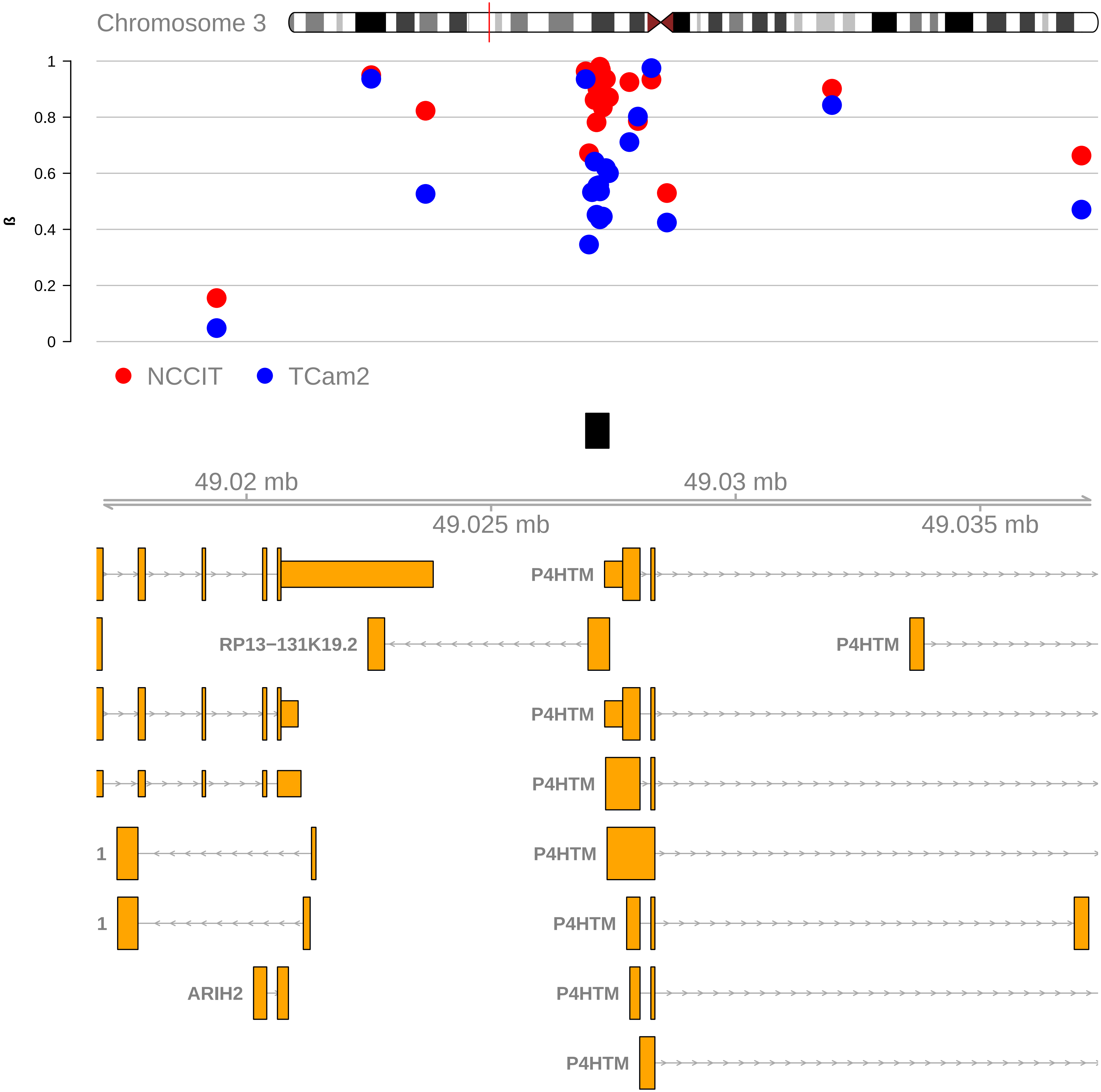
Chromosome 3
1
0.8
0.6
0.4
0.2
0
ß
NCCIT
TCam2
49.02 mb
49.03 mb
49.025 mb
49.035 mb
P4HTM
RP13−131K19.2
P4HTM
P4HTM
P4HTM
1
P4HTM
1
P4HTM
ARIH2
P4HTM
P4HTM
